# Comparison of the Effects of Mucosa Tissue Healing with Chlorhexidine Digluconate and Choline Salicylate in Patients Wearing a Removable Prosthetic Restoration—A RCT

**DOI:** 10.3390/clinpract14060184

**Published:** 2024-11-04

**Authors:** Barbara Sekuła-Kamińska, Aleksandra Nitecka-Buchta, Mateusz Wojciechowski, Małgorzata Skucha-Nowak, Małgorzata Rymarczyk, Stefan Baron

**Affiliations:** 1 Department of Temporomandibular Disorders, Medical University of Silesia in Katowice, Traugutta Sq. 2, 41-800 Zabrze, Poland; barbara.sekula@sum.edu.pl (B.S.-K.); d201064@365.sum.edu.pl (M.W.); malgryma@interia.pl (M.R.); sbaron@sum.edu.pl (S.B.); 2 Unit of Dental Propedeutics, Medical University of Silesia in Katowice, Dworcowy Sq. 3, 41-800 Zabrze, Poland; mskucha-nowak@sum.edu.pl

**Keywords:** chlorhexidine, choline salicylate, oral mucosa, decubitus, removable partial dentures

## Abstract

Background and Objectives: A randomized, double-blind clinical trial was conducted based on the CONSORT study protocol for randomized clinical trials (NCT06531720) to compare the effectiveness of oral mucosa healing properties of 0.2% chlorhexidine digluconate (CHX) and 8.7% choline salicylate (CHS), as well as a control group (CON) with no intervention, in patients with delivered partial removable dentures (PRDs). Materials and Methods: Patients (n = 27) who were enrolled in the study were healthy subjects according to the inclusion/exclusion criteria, and they received new PRDs to complement Kennedy’s class III and IV deficiencies. During the process of adaptation to new prosthetic restorations, OMLs were formed and treated with one of two selected preparations, either CHX = 0.2% or CHS = 8.7%, in relation to the control group (CON). The wound surface area (WSA) (mm^2^) was measured on repeatable intraoral images taken in accordance with the examination protocol on the first control visit on day 1, day 3, day 7, day 10, and day 14 with the assistance of computer software. Results: There were no statistically significant differences between groups. The fastest effect of WSA complete reduction was observed in the CHX group after 7 days (WAS = 0.78, SD = 1.18) in comparison to CHS = 10 days (WAS = 0.44, SD = 0.90) and CON = 14 days (WAS = 0.22, SD = 0.67). The decrease in the WSA after 7 days of observation was 85.1% in the CHX group, 70.1% in the CHS group, and 59.2% in the CON group. Conclusions: The WSA decreased most rapidly after 7 days of treatment with 0.2% chlorhexidine digluconate (CHX), slightly more slowly after 10 days of treatment with 8.7% choline salicylate (CHS), and relatively most slowly in the CON group, who were not treated with any topical medication after 14 days. Oral mucosa lesions (OMLs) therapy during the process of adaptation to new removable prosthetic restorations is a very important element supporting the whole process. Topical medications containing 0.2% chlorhexidine digluconate are indicated as adjunctive therapy in the process of the supportive treatment and disinfection of oral mucosa lesions. However, this does not release the dentist from liability for the careful adjustment of the removable prosthetic restoration.

## 1. Introduction

The efficacy of oral topical medication in the healing and regeneration of oral mucosa lesions (OMLs) caused by removable dental prostheses has been analyzed. The healing process of the mucous membrane is an extremely important factor in the adaptation to new prosthetic restorations and significantly affects the quality of life of patients using these restorations [1,2,3]. Quality of life in patients using RDP is much lower than that of dentate patients and lower than that of patients equipped with implant-based restorations [3].

Removable partial dentures (RPDs) are prosthetic reconstruction devices designed and manufactured for partially edentulous patients. Long-term RPDs are common for patients with no appropriate bone volume, structure, or quality who cannot be treated with dental implants. RPDs are a removable prosthesis, which means that they should be removed from the oral cavity, cleaned, and reinserted several times a day. Especially in the initial period after their implementation, painful OMLs may develop under the denture plate. The task of a specialist prosthodontist is also to treat these OMLs. In our study, removable dentures covering an oral mucosa lesion were adjusted during the control visit. Oral cavity mucosa is characterized by fast wound healing and a scarless outcome as compared to skin wounds [4]. Fast healing of this OML plays an important role in preventing microbes’ invasion, avoiding the process of chronic infection. The body’s natural defense reactions help to protect it against the harmful effects of the unfavorable oral cavity environment [2]. The healing cascade involves subsequent processes such as inflammation, cell proliferation, the angiogenesis of blood vessels, and collagen synthesis. The healing of oral mucosa lesions is severely affected by the aging process [5]. The majority of patients using RDP are middle-aged and elderly.

Topical anti-inflammatory medications are recommended for patients who developed OMLs during the adaptation period to new removable dentures thanks to their antibacterial and healing properties. The efficacy of these agents has been evaluated based on a comparison of the surface area of oral lesions before and after treatment. In this study, the authors compared the efficacy of two topical oral medications in the healing of oral mucosa lesions and the antibacterial properties. One of the medications contained 0.2% chlorhexidine digluconate and the other 8.7% choline salicylate as active ingredients and an ethanol-based formulation.

Chlorhexidine (CHX) is a biguanide frequently used in oral healthcare because of its antibacterial features, commonly as mouthwashes, dental gels, or oral sprays. The concentration of chlorhexidine varies depending on the preparation used [6]. Various concentrations are available, from 0.12% to 0.5% CHX, depending on the indications, severity of symptoms, and duration of use of the preparation.

Choline salicylate (CHS) diminishes inflammatory reactions because it is a non-steroidal anti-inflammatory drug (NSAID) derived from salicylic acid. By inhibiting prostaglandin synthesis, it shows anti-inflammatory, analgesic, and antipyretic effects on the hypothalamic heat control center [7].

There is a variety of topical mucosa medications recommended for patients developing oral mucosa lesions during the period of adaptation to new dentures. These products are based on different active ingredients, such as gel, cream, and solution, and have many different functional properties [8]. Nanotechnologies are offering mucoadhesive gel systems for buccal drug delivery that may be very helpful in delivering active substances to the damaged region. The most popular are chlorhexidine, allantoin, local anesthetics, grapefruit, sage, chamomile baicalensis root extracts, and calf blood dialysate. Due to the high diversity of ingredients, it is a major problem for both clinicians and patients to make a proper choice in a given clinical situation [9]. Hyaluronic acid is also recommended for use on oral mucosa wounds during the healing process [10].

## 2. Materials and Methods

The efficacy of oral topical medications in aiding the regeneration and healing of oral soft mucosa, caused by the use of RDP, was analyzed. The evaluation of the efficacy of these agents was based on a comparison of the area of OMLs before and after the treatment (WSA [mm^2^]). The aim of this study was to compare the effectiveness of the OM healing properties of different preparations, CHX = 0.2% chlorhexidine digluconate, CHS = 8.7% choline salicylate, and CON = control group, in patients with delivered removable dentures during the adaptation period.

**Figure 1:**
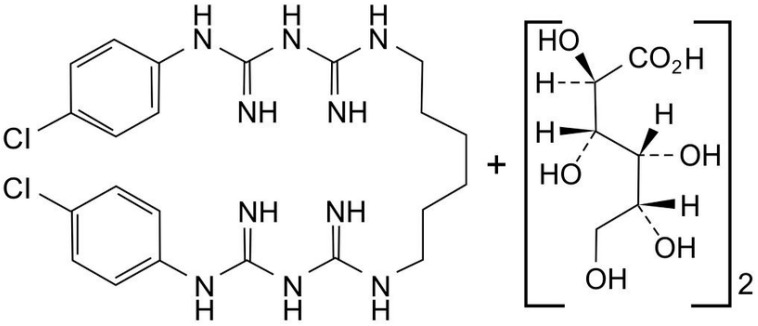
Chlorhexidine digluconate chemical structure.

**Figure 2:**
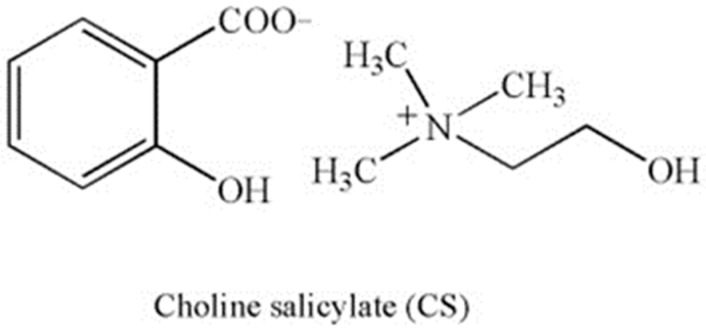
Choline salicylate chemical structure.

This study was designed as a double-blind clinical parallel trial to evaluate healing after the application of a topical medication containing 0.2% CHX (Figure 1), 8.7% CHS (Figure 2), and a control group without any topical medication (CON).

**Figure 3:**
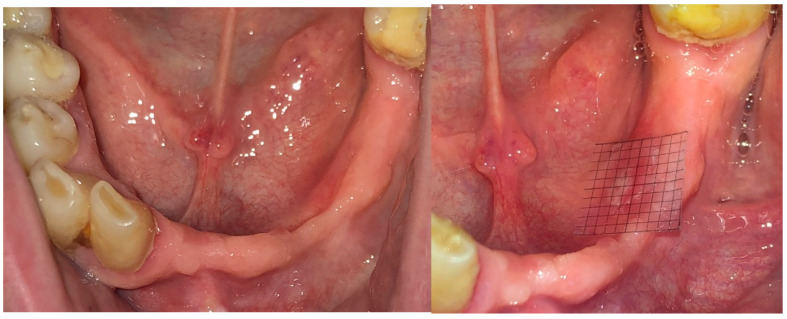
Method of measuring the size of the WAS (wound area surface) in [mm^2^] during control visit of removable partial denture adjustment.

A randomized, double-blind clinical trial was conducted based on the CONSORT study protocol for randomized clinical trials (NCT06531720) [11,12]. All the recommendations of the CONSORT checklist were followed, and a checklist was added as an attachment to this article (see additional materials). Patients who qualified for the study (n = 27) were randomly selected from the patients treated at the Prosthodontics and TMD Clinic, Medical University of Silesia in Zabrze, Poland. A specialist in prosthetic dentistry—BSK—enrolled patients to the study, ANB and MW performed control visits and measured OMLs by taking intraoral photos (Figure 3), and SB was responsible for the randomization process and the blinding of the patient’s data. MSN was responsible for content supervision. Neither patients nor dental practitioners knew what kind of topical medication was given to the patients by dental nurses in disposable syringes. Patients were using the topical medication at home. Two parallel groups of patients were treated with CHX and CHS compared to the CON group. The obtained data were subjected to statistical analysis with Statistica 12.0 Software (Stat Soft, Poland) and Microsoft Excel Spreadsheets. 

### 2.1. Group Characteristics

Patients enrolled in this study were qualified for prosthetic rehabilitation with removable partial dentures, according to Kennedy’s classification class III and IV [13]. A total of 132 patients, who received removable dentures between 1 January 2023 and 31 December 2023 in the Prosthodontics and TMD Clinic, Medical University of Silesia in Zabrze, Poland, were enrolled in the study. Only 27 patients with OMLs met all the inclusion criteria, and they were divided into 3 groups: oral mucosa lesions treated with topical use of 0.2% chlorhexidine digluconate: CHX = 9, 8.7% choline salicylate: CHS = 8, and CON = 9. Participants were enrolled in the study after meeting the inclusion and exclusion criteria (Table 1).

The clinician in charge of the randomization process assigned the patients to one of the three study groups: CHX, CHS, or CON (Table 2). Randomization was conducted by drawing a group number (CHX, CHS, or CON) from the unmarked envelope during the first appointment (0 day). Randomization was conducted by SB, regardless of age and sex.

Patients gave written consent to participate in the study and to use the study data, excluding sensitive personal data.

Patients were asked to use topical medication 3 times a day, applying the size of a pea on the oral mucosa lesion. Participants in groups CHX and CHS were instructed not to eat or drink for 1 h following the application of the medication because of its functional and antibacterial properties. Patients were asked to wear new dentures between control visits, even when they felt discomfort while using them. Patients in the control group were not using any topical medication. According to randomization, patients suffering from oral mucosa lesions (OMLs) were divided into three groups:

- Group I: oral mucosa lesions treated with 0.2% CHX topical use;
- Group II: oral mucosa lesions treated with 8.7% CHS topical use;
- Group III: oral mucosa lesions as control group (CON) with no specific OML treatment.

None of the patients were informed about the topical medication they were using. None of the practitioners involved in this study were informed of which substance was used in the therapy. Only SB collected the data of patients and group allocation.

Only patients with an OML in the area of the anterior vestibular surface of the maxilla alveolar process and the vestibular surface of the anterior alveolar region of the mandible were enrolled in the study. OMLs were properly photographed and documented before performing the graphical analysis in Photo for Windows Software. Using a transparent millimeter grid superimposed on the OML, the wound surface area (WSA) was measured in mm^2^. A very precise measurement of the WSA was possible thanks to the use of a millimeter scale on the digital photo image. Every, even those that were partially filled, square was included in the area of the OML. Graphical analysis using Photo for Windows Software consisted of adding all the squares within which the OML was observed. All the intraoral images of the OMLs were taken in reproducible conditions (ambient light, lens and camera settings, distance 5 cm). During the first control visit (0 day) when the new denture was controlled and adjusted, the occurrence of the OML and its site were assessed. RPDs were adjusted with the aid of a soft A-silicone impression material Elite HD+ Light Normal (Zhermack GmbH, Marl, Germany). In the area of excessive pressure on the mucosa, the A-silicone material was thinned out. After taking a functional occlusal impression, the area of excessive pressure was visualized, and adjustment of the denture was carried out with a sintered carbide drill and prosthetic micromotor. The procedure was carried out until an equal layer of Elite impression material compound was obtained. Occlusion was also checked with Bausch occlusal articulating foil at 40 µm, and correction was performed if needed.

**Figure 4:**
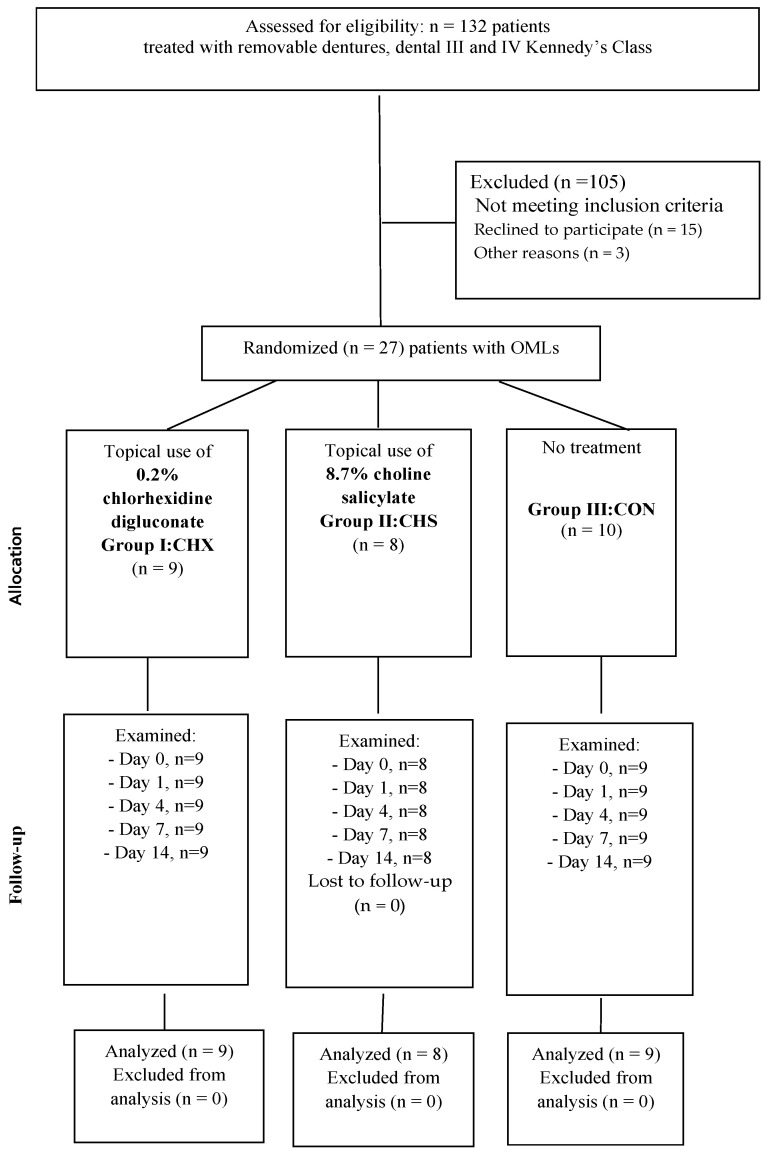
Flowchart of the study.

This study was performed according to the following flowchart (Figure 4).

### 2.2. Statistical Methods

As part of the statistical analysis of the results of this study, comparisons of the WSA between the study groups on consecutive days of observation were made first. The applicability of the parametric test—one-way ANOVA—was checked by verifying the normality of the distribution using the Shapiro–Wilk test. In those cases where this verification was positive in each group, a parametric test was used; in cases where this wasn’t positive, a non-parametric test (Kruskal–Wallis) was used. A comparison of the WSA between the CHX group and the CON group at the end of the observation period was made using the Mann–Whitney test. To assess the significance of changes in the WSA over time, in each group separately, the non-parametric Friedman test was used. In cases where significance was obtained, the Conover post hoc test was used, with which the significance of differences over consecutive days was assessed on a one-to-one basis. Calculations and graphical elaboration of the results were carried out using an Excel (Microsoft, Albuquerque, NM, USA) spreadsheet. *p* < 0.05—level of significance—was considered when formulating conclusions as statistically significant. All the materials needed to conduct statistical analysis were made available by the authors. The authors of this work will provide the materials of statistical analysis upon special request.

### 2.3. Sample Size Calculation

A one-way ANOVA Calculator was used to estimate the sample size in the research. The level of significance was *p*-value = 0.05, outliers were included, the medium size effect was chosen, and the f-type effect was chosen. Sample size was calculated on the basis of data entered directly into the ANOVA Calculator (www.statskingdom.com, 25 October 2024). Normality was assessed, *p*-value = 0.835 was calculated between groups, and the difference between samples was not big enough to be statistically significant. The test statistic F equals 0.18, which is in the 95% region of acceptance. The observed effect size was medium (0.2). The test power was low: 0.093, so the test could not reject an incorrect h_0_. This should be improved by using a larger sample size. Determining new features of the research should be carried out before collecting data for the research, but it was not easy to estimate the number of patients enrolled in the study who were attending control visits. The assumption was checked based on the Shapiro–Wilk test, and it was stated that the sample size needed to be greater, with at least 30 participants in each group. This research was planned as a pilot study; further evaluation of the effects of the CHX, CHS, and CON groups is needed.

## 3. Results

**Figure 5:**
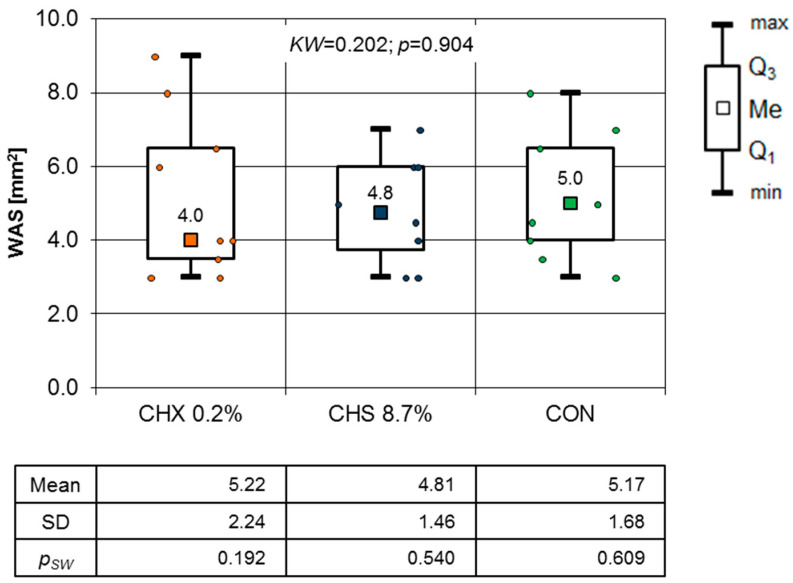
Area of OML on the first day of follow-up (Kruskal–Wallis test results).

The mean age of the patients was 65.4 years SD +/−10.7 years (Table 2). There were 16 male and 11 female patients; there were no statistical differences between groups (*p* < 0.05). The comparison of the differences in the WSA of the oral mucosa lesion during the first follow-up visit between all three groups is shown in Figure 5. The Kruskal–Wallis test results (*p* = 0.904) indicate that there were no statistically significant differences between groups. This was confirmed by the one-way ANOVA test results (F = 0.122; *p* = 0.886).

**Figure 6:**
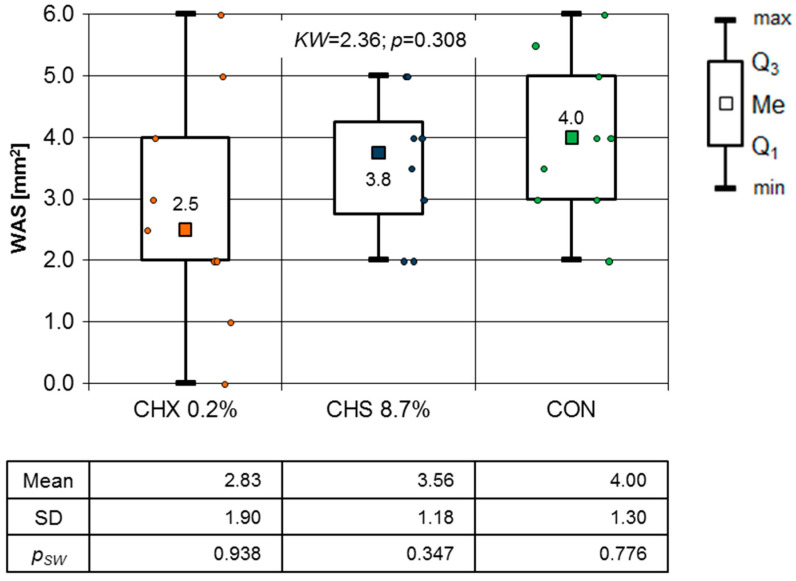
WSA mm^2^ on the third day of follow-up (Kruskal–Wallis test results).

In the comparison of the WSA on the day 3 follow-up visit, no statistically significant differences were found based on the results of the Kruskal–Wallis test (*p* = 0.308) (Figure 6). The same conclusion was proved by the one-way ANOVA test results (F = 1.375; *p* = 0.273).

**Figure 7:**
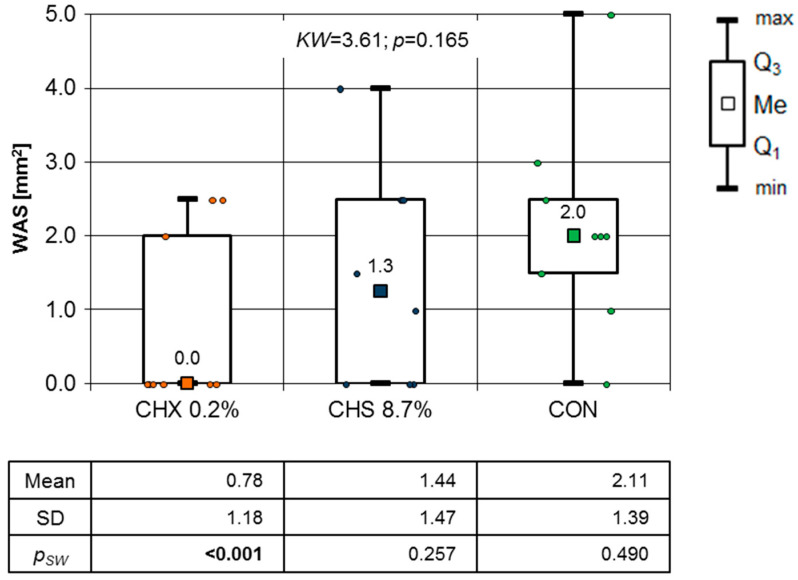
WSA mm^2^ at seventh day of follow-up (Kruskal–Wallis test results).

After seven days of observation, the comparison of the WSA showed no statistically significant differences. This was confirmed by the results of the Kruskal–Wallis test (*p* = 0.165). From that day onwards, only the aforementioned test was used. In the group using the CHX medication, the decubitus surface area distribution deviated significantly from the normal distribution (Shapiro–Wilk test results *p* < 0.001) (Figure 7). The difference in the WSA in mm^2^, expressed as a percentage on the 7th day of healing, was as follows: CHX 7 day = 85.1%, CHS 7 day = 70.1%, and CON 7 day = 59.19% (Table 3).

**Figure 8:**
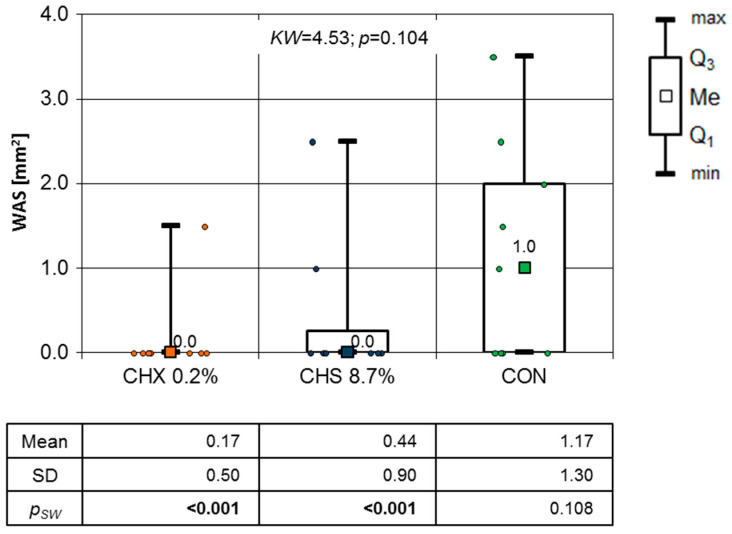
WSA mm^2^ at tenth day of follow-up (Kruskal–Wallis test results).

In further observations, the results of the Shapiro–Wilk test excluded the use of a parametric test (one-way ANOVA). Because of that fact, Figure 7 and Figure 8 present the results of a non-parametric test (Kruskal–Wallis test) to standardize inference.

**Figure 9:**
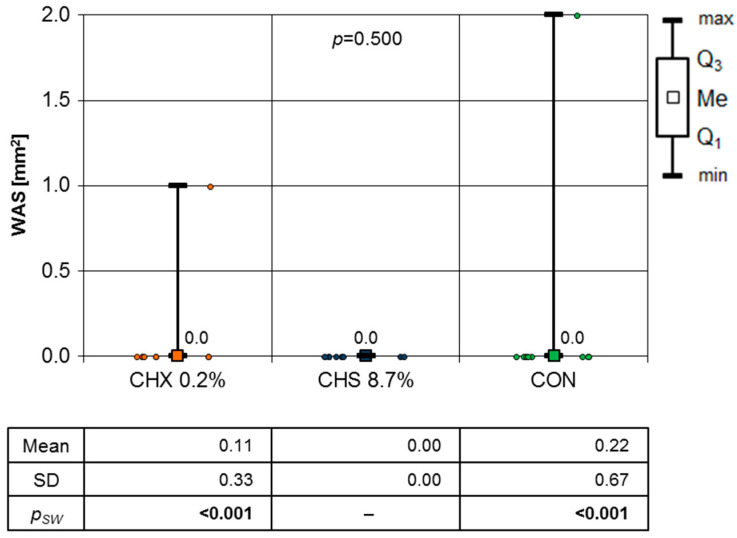
WAS mm^2^ at fourteenth day of follow-up (Mann–Whitney test results).

The results of the Mann–Whitney test are presented in Figure 9, and they were used to compare the WSA in the CHX group and the CON group. Hence, it was necessary to use the Mann–Whitney test, of which the results indicate that there was no statistically significant difference (*p* = 0.500).

**Figure 10:**
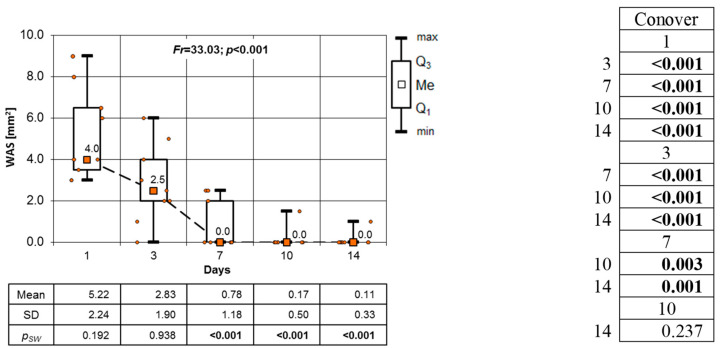
Changes in the WSA mm^2^ of OML in CHX group on subsequent days of observation (results of the Friedman test and Conover’s post hoc test).

**Figure 11:**
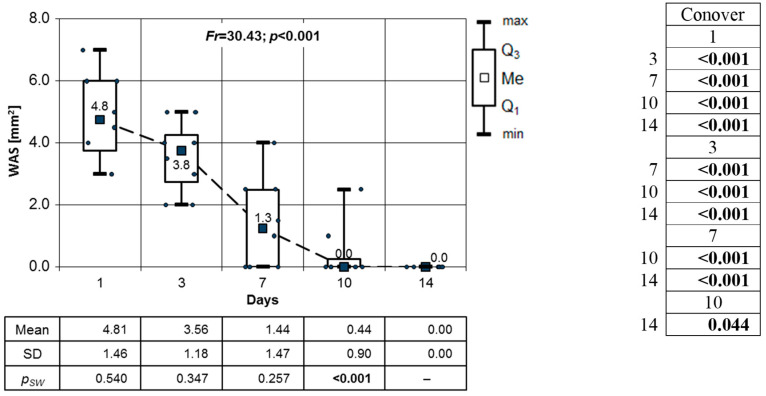
Changes in the WSA mm^2^ of OML in CHS group on consecutive days of observation (results of Friedman’s test and Conover’s post hoc test).

**Figure 12:**
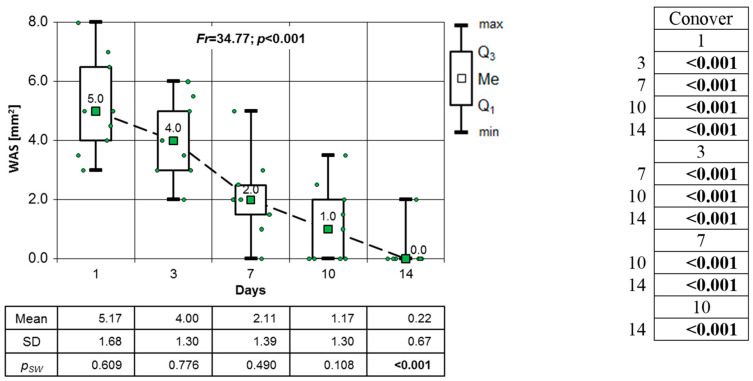
Changes in the WSA mm^2^ of OML in the control group on consecutive days of observation (results of the Friedman test and Conover’s post hoc test).

Figure 10, Figure 11 and Figure 12 show the results of the Friedman test used to assess changes in the WSA of the OML over time in each group separately. The results of the test for the CHX group show a very significant decrease in the WAS of the OML over the entire observation period.

The high (*p* < 0.01) and very high (*p* < 0.001) significance of these changes is presented by the results of the Conover post hoc test. The only exception is the variation in the WAS between days 10 and 14. The lack of significance of this difference is due to the fact that on day 10, the OML was already fully healed. In the CHS group, the Friedman test results show a major decrease in the WSA in the OMLs on consecutive days of observation. In the analysis, data from the 14th day of the follow-up were omitted since all patients in this group showed no sign of OMLs (Figure 11). All noted changes in the WSA are highly statistically significant (*p* < 0.001).

In the CON group, as well as in groups where patients were prescribed a topical medication, a very significant decrease in the WSA was observed (Friedman test result *p* < 0.001; post hoc test results *p* < 0.001) (Figure 12). It was not until the 14th day of observation that the lesion was almost fully healed.

**Figure 13:**
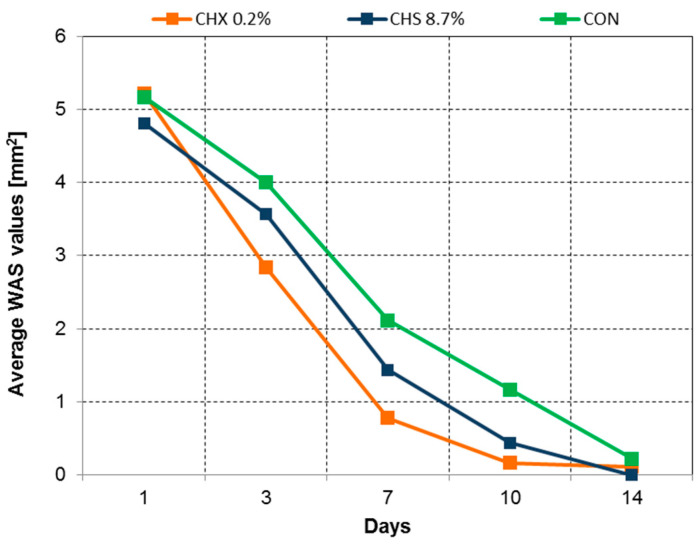
Changes in the mean WSA mm^2^ on successive days of observation for CHX, CHS, and CON groups. Ultimately, after two weeks, the WSA in each of the study groups reached virtually zero.

To illustrate the OML healing process in this study, a graph of the mean WSA values over the entire observation period is presented. Although there were no significant differences between consecutive days (Figure 5, Figure 6, Figure 7, Figure 8 and Figure 9), it is evident that the WSA decreased most rapidly after treatment with 0.2% chlorhexidine digluconate in the CHX group, slightly more slowly after treatment with 8.7% choline salicylate in the CHS group, and relatively most slowly in the CON group, where members of which were not treated with any topical medication (Figure 13).

As summarized in Table 3, the highest percentage reduction in the WSA occurred after 7 days in the CHX group; although the differences were not statistically significant, this may be due to the small size of the study groups (Table 3). The WSA after 7 days of observation was calculated as an 85.1% reduction in the CHX group, a 70.1% reduction in the CHS group, and a 59.2% reduction in the CON group. The main limitation of this research was the small number of participants meeting the inclusion criteria for this study.

## 4. Discussion

Chlorhexidine digluconate is often used in hospitals and emergency medicine departments when treating oral mucosal lesions. In patients with poor oral hygiene or after surgery procedures in the oral cavity, they are advised to use CHX 2% as a mouth rinse or spray. Solderer in his systematic review noted that CHX may be a valuable preventive tool to use immediately after surgery during the time period in which oral hygiene capacity is compromised [14]. To reduce the side effects of CHX and maintain comparable clinical effects, rinsing with 0.12% showed the most promising results so far. Compared to our study, the CHX concentration of 0.2% is higher than that suggested for post-surgery oral hygiene suggestions. After approximately 4 weeks of continuous CHX use, tooth staining, calculus build up, transient taste disturbance, and effects on the oral mucosa should alleviate [15]. Rinsing the oral cavity with a 0.5% concentration of CHX is effective for oral mucosa healing, but some authors have observed that when the concentration of CHX is increased, a delay in wound healing can occur. Frequent and intensive rinsing with high concentrations of CHX may develop a delay in and the disturbance of wound healing in humans [16]. According to Gurgan, 0.2% alcohol-free CHX for 1 week caused more irritation to the oral mucosa, a greater burning sensation, and increased altered taste perception compared to the placebo rinse [17]. In our research, we did not observe any side effects of CHX intraoral use; topical medication gel for OML treatment did not cause unfavorable side effects.

CHX 0.2% is a popular antiseptic oral cavity therapy preparation that has been used for soft tissue inflammation for many years [18]. Hamp et al. have analyzed gingival wounds healing in dogs. OMLs treated with saline regenerated extensively with greater inflammation symptoms compared to OMLs treated with 0.2% CHX, which healed with minor signs of inflammation [19]. According to Pilloni, the production of collagen fibers, cell proliferation, and apoptosis in CHX-treated OMLs was more intensive [19], and collagen deposition was increased. A better effect on the scar pattern healing of soft tissues was also observed after the CHX application. The fibrotic transformation of CHX is very desirable in the case of the healing of the pressure of OMLs. Under the denture base, soft tissues adapts faster to the increased load. Broad-spectrum antimicrobial CHX activity prevents biofilm formation and improves OM healing. According to Graziani [20], CHX protects against re-contamination of the wound and accelerates healing, as was observed in our study.

CHX has not only advantages; there are some disadvantages: here are some publications about the toxicity of 2% CHX. A high incidence of oral mucosal lesions was observed after 2% CHX, but 0.2% or 0.12% was less harmful to the oral mucosa [8,21]. That is why in our research study, a 0.12% topical preparation of gel was used. According to Bassetti, better results in OML healing are achieved after rinsing the wound with Ringer solution compared to 0.5% CHX [22]. In this study, we achieved better results in patients using topical medication with 0.2% CHX because of the functional, antibacterial, and physical properties of chlorhexidine.

Madrazo-Jiménez published a research study with gel composed of chitosan, 0.2% chlorhexidine, allantoin, and dexpanthenol which improved wound healing after the surgical extraction of impacted lower third molars [9]. Also, a very interesting article was published by Baus-Domínguez, designed with a multispecies biofilm model of intraoral bacteria species cultured with CHX 0.2% in a bioadhesive gel and cymenol 0.1% [23]. The great effectiveness of the antimicrobial action and high penetrability in the biofilm were observed thanks to the cymenol 0.1% addition [23].

However, Gurgan has observed more irritation in OM after CHX therapy compared with a placebo [24]. Kalyani used chlorhexidine and metronidazole gel for wound healing after incision in the oral cavity [25]. The study sites showed better wound healing and decreased postoperative inflammation. There was a statistically significant decrease in postoperative pain in the study site in the metronidazole–chlorhexidine gel group [25]. The presence of alcohol may increase the effectiveness of CHX in early wound healing according to Gkatzonis [26].

Berchier published his research comparing 0.12% and 0.2% CHX concerning the effect on gingival inflammation [27]. With respect to plaque inhibition, the results showed a small but significant difference in favor of the 0.2% CHX concentration. However, the clinical relevance of this difference is probably negligible. Gurav has observed that the use of CHX bioadhesive agents can improve the healing therapy of OMLs [8].

In this research study, topical preparations containing choline salicylate (CHS) were prescribed to participants. Choline salicylate (CHS) is used in recurrent aphthous ulceration for tissue decontamination, pain reduction, and healing promotion [26]. CHS should be used with caution in subjects with renal failure, stomach ulcers, anemia, salicylates intolerance, and coagulation disorders [7]. In this research study, no side effects were observed after the topical administration of CHS. An allergic reaction can be observed to choline salicylate when used as a component of oral mucosa lesion healing gel. The authors of this research study did not observe any unfavorable reactions to CHS. This active substance, in those who are sensitive to salicylate, can provoke a Stevens–Johnson syndrome [27], so a special medical interview should be perform prior to the treatment. A simple allergic test can be performed prior to intraoral use. Wróblewska, in her article [7], deemed choline salicylate preparation for OML therapy to be a practically stable and chemically neutral process, so it is recommended in OML patients [7]. To sum up, it can be said that chlorhexidine is a factor that has a beneficial effect on the healing of tissues in the oral cavity, especially in patients with OMLs. More research in this field is needed, with at least 30 participants in each group. This research was planned as a pilot study; further evaluation of the effects of the CHX, CHS, and CON groups is needed.

## 5. Conclusions

Within the limits of this clinical research, based on the small sample size, we have observed improvements in the healing of oral mucosa lesions after the use of topical medication composed of 0.2% chlorhexidine digluconate gel compared to 8.7% choline salicylate gel and a control group with no intervention. The effectiveness of oral mucosa healing properties, measured with the WAS (wound surface area, mm^2^) parameter, was most effectively reduced in the CHX group—85.1% in 7 days of the therapy. During the same time, the WAS reduction in the CHS group was 70.1% and 59.2% in the CON group. Patients treated with partial removable dentures (PRDs) should receive recommendations of an additional treatment with topical medications to help reduce pain and accelerate the healing of oral mucosa lesions under the denture base. Such activities have a positive impact on cooperation with patients, improve patient comfort, and accelerate adaptation to new prosthetic restorations.

## Figures and Tables

**Table 1:** Inclusion and exclusion criteria for patients participating in the research study.

Inclusion Criteria	Exclusion Criteria
-Oral mucosa lesion (OML) as a result of new denture adaptation	-Systemic conditions affecting wound healing: diabetes
-RDP partial dentures III and IV	-Prosthetic stomatitis according to Newton’s classification
-RDP prostheses delivered for the patient during the period of the study	-Parafunctional habits, bruxism
-Patient consent	-Autoimmune diseases

**Table 2:** Characteristics of the CHX, CHS, and CON groups.

	CHX GROUP	CHS GROUP	CON GROUP
AVERAGE AGE	63.2 years SD +/−7.9	67 years SD +/−5.2	69 years SD +/−9.1
GENDER M/F	6 male, 3 female	5 male, 3 female	5 male, 5 female

**Table 3:** Reduction (%) in WSA mm^2^ in CHX, CHS, and CON groups after 7 days.

	CHX GROUP	CHS GROUP	CON GROUP
AVERAGE WSA day 0 [mm^2^]	5.22	4.81	5.17
AVERAGE WSA day 7 [mm^2^]	0.78	1.44	2.11
% REDUCTION	85.1%	70.1%	59.2%

## Data Availability

The original contributions presented in this study are included in the article; further inquiries can be directed to the corresponding author.
